# OxyR contributes to virulence of *Acidovorax citrulli* by regulating anti-oxidative stress and expression of flagellin FliC and type IV pili PilA

**DOI:** 10.3389/fmicb.2022.977281

**Published:** 2022-09-20

**Authors:** Jianan Wang, Jun Liu, Yuqiang Zhao, Minghui Sun, Guixu Yu, Jiaqin Fan, Yanli Tian, Baishi Hu

**Affiliations:** ^1^College of Plant Protection and Key Laboratory of Integrated Management of Crop Diseases and Pests, Nanjing Agricultural University, Nanjing, China; ^2^Institute of Plant Protection and Soil Fertilizer, Hubei Academy of Agricultural Sciences, Wuhan, China; ^3^Institute of Botany, Jiangsu Province and Chinese Academy of Sciences (Nanjing Botanical Garden Mem. Sun Yat-Sen), Nanjing, China; ^4^Laboratory of Bacteriology, Department of Plant Pathology, Nanjing Agricultural University, Nanjing, China

**Keywords:** *Acidovorax citrulli*, OxyR, oxidative stress, virulence traits, virulence

## Abstract

In many bacteria, OxyR acts as a transcriptional regulator that facilitates infection *via* degrading hydrogen peroxide (H_2_O_2_) generated by the host defense response. Previous studies showed that OxyR also plays an important role in regulating biofilm formation, cell motility, pili relate-genes expression, and surface polysaccharide production. However, the role of OxyR has not been determined in *Acidovorax citrulli* strain xjl12. In the current study, the qRT-PCR and western blot assays revealed that the expression level of *oxyR* was significantly induced by H_2_O_2_. The *oxyR* deletion mutant of *A. citrulli* was significantly impaired bacterial tolerance to oxidative stress and reduced catalase (CAT) activity. In addition, *oxyR* mutant resulted in reduced swimming motility, twitching motility, biofilm formation, virulence, and bacterial growth in *planta* by significantly affecting flagellin and type IV pili-related gene (*fliC* and *pilA*) expression. The qRT-PCR assays and western blot revealed that OxyR positively regulated the expression of *fliC* and *pilA.* Furthermore, bacterial one-hybrid assay demonstrated that OxyR directly affected *pilA* and *fliC* promoter. Through bacterial two-hybrid assay, it was found that OxyR can directly interact with PilA and FliC. These results suggest that OxyR plays a major role in the regulating of a variety of virulence traits, and provide a foundation for future research on the global effects of OxyR in *A. citrulli*.

## Introduction

The Gram-negative bacterium *Acidovorax citrulli* is the causal agent of bacterial fruit blotch (BFB; Schaad et al., 1978, 2008; Willems et al., 1992), a threatening disease of cucurbit crop species worldwide (Schaad et al., 2003; Burdman and Walcott, 2012). According to Hopkins and Thompson (2002), the bacterium is transmitted and spread by infected seeds, which are the primary inoculum sources for BFB outbreaks. Seed treatments can effectively reduce the spread of disease but often fail to eradicate pathogens in seeds (Dutta et al., 2012). Therefore, understanding the mechanisms of host-pathogen interactions is of great importance for effective BFB management. The interaction between plants and pathogens is that pathogens infect host cells with a variety of virulence traits to acquire nutrients and water for survival and development, while plants prevent the invasion of pathogens through defense responses (Chisholm et al., 2006; Jones and Dangl, 2006; Kunkel and Chen, 2006). *A. citrulli* utilizes multiple virulence traits to infect plant cells, e.g., type II secretion system (T2SS; Johnson, 2010), type III secretion system (T3SS; Ren et al., 2009; Johnson et al., 2011), type VI secretion system (T6SS; Tian et al., 2014), type IV pili (T4P; Bahar et al., 2009), polar flagella (Bahar et al., 2011), and quorum sensing (QS; Wang et al., 2016). Therefore, the hallmark of successful pathogen infection is the ability to effectively inhibit the immune systems of plants (Kunkel and Chen, 2006).

The immune system of plants against pathogens is primarily based on two immune defense mechanisms. Plant cells can detect conserved molecules of the pathogen called pathogen (microbe)-associated molecular patterns (PAMPs/MAMPs) using pattern recognition receptors (PRRs) to inhibit the survival of the pathogen (Zipfel, 2008, 2009). This response is collectively termed PAMP-triggered immunity (PTI). However, some pathogens have evolved the ability to evade or inhibit PTI by secreting a protein called an effector (Block and Alfano, 2011). Thus, plants have evolved a secondary defense called effector-triggered immunity (ETI) to specifically recognize effectors secreted by pathogens (Dodds and Rathjen, 2010). Plant immune responses to pathogens infection include oxidative burst, rapid changes in gene expression, and cell wall reinforcement (Zipfel, 2008; Yu et al., 2016).

The oxidative burst involves the production of reactive oxygen species (ROS), including hydrogen peroxide (H_2_O_2_), superoxide (O_2_^−^), and hydroxyl radical (HO˙). ROS plays an important role in plant defense, such as acting as signaling molecules that form oxidative cross-linkages in plant cell walls to prevent pathogen invasion (Torres, 2010). Therefore, anti-oxidative activity is essential for the successful growth and survival of pathogens under environmental stresses (Hébrard et al., 2009). OxyR is a DNA-binding transcription factor that not only acts as an activator of genes encoding peroxide-detoxifying enzymes, but also against oxidative stress (Jo et al., 2015; Ishiga and Ichinose, 2016). OxyR regulates many genes concerned with defense against hydrogen peroxide (H_2_O_2_), e.g., *katA* and *katB* (encoding catalases A and B), *katG* (encoding hydroperoxidase I), *dps* (encoding DNA-binding protein from starved cells), and a*hpCF* (encoding an alkyl hydroperoxide reductase; Ochsner et al., 2000; Hishinuma et al., 2006; Italiani et al., 2011; Xia et al., 2017). In addition, OxyR also plays a critical role in regulating biofilm formation, pili relate-genes expression, mucosal colonization (Hennequin and Forestier, 2009), and surface polysaccharide production (Shin et al., 2020) in pathogenic bacteria.

At the moment, the role of OxyR in *A. citrulli* is still unclear. In this study, we identified *oxyR* (*Aave_0594*) from the AAC00-1 genome (GenBank accession number NC_008752) to functionally characterize OxyR in *A. citrulli*. We evaluated the regulatory mechanism and function of OxyR in oxidative stress resistance, as well as its contribution to host virulence. The qRT-PCR assay demonstrated that OxyR regulates the oxidative stress-related gene of *catB*(*Aave_3137*) and *ahpC*(*Aave_1375*). In addition, qRT-PCR, western blot, bacterial one-hybrid, bacterial two-hybrid, and phenotype assay showed that *oxyR* affects the twitching motility, biofilm formation, and swimming motility by positively regulating the expression of *pilA* (*Aave_4679*) and *fliC* (*Aave_4400*) genes and interaction in *A. citrulli*. This study revealed that OxyR is one of the essential virulence factors that supports *A. citrulli* pathogenesis in melon.

## Materials and methods

### Bacterial strains and growth conditions

*A. citrulli* wild-type and its derived mutants were routinely cultured in Luria-Bertani (LB) medium at 28°C with shaking at 220 rpm, with or without 1.5% (wt/vol) agar (Sambrook et al., 1989). All *Escherichia coli* strains were cultured in LB medium at 37°C. The optical density of cell suspensions at 600 nm was used to track the growth of bacteria. All strains were stored at −80°C for long-term storage. Following final concentrations of antibiotics were provided: 100 ug/ml rifamycin (Rif), 50 ug/ml kanamycin (Km) and 100 ug/ml gentamicin (Gm). Supplementary Table S1 lists all of the bacterial strains and vectors used in this study.

### Construction and complementation of deletion mutants of *Acidovorax citrulli*

Deletion mutations of *oxyR*, *catB*, *ahpC*, *pilA,* and *fliC* were generated using homologous recombination in *A. citrulli*, as described previously (Liu et al., 2019). Briefly, based on the AAC00-1 genome sequence, two flanking regions (upstream and downstream) of target genes were generated by PCR amplified using the primer pairs and cloned into pEX18GM. A Km fragment was placed into the middle of the two fragments to create the recombinant vector in order to expedite the screening of mutants. This recombinant vector was transformed into *A. citrulli* xjl12. On LB plates with 10% (wt/vol) sucrose, Rif (100 mg/ml), and Km (50 mg/ml), we successfully picked the transformed colonies, which were further confirmed by PCR amplification using primers F1 and R2 (data not shown). A proven mutant was chosen for additional investigation.

The online promoter prediction website^1^ was used to predict the promoter of genes for complementing the deletion mutant of *A. citrulli*. A pair of specific primers, comp-F/R, was created to amplify a region including the gene and its predicted promoter site. The amplicon was subcloned into pMD19-T and this fragment was sequenced to check for base mutations. Following the appropriate restriction enzyme digestion, the fragment was cloned into the expression vector pBBR1MCS-5 (Kovach et al., 1995). Then, the recombinant vector was transferred into gene mutants. Transformants were screened on LB agar plates with Gm (50 ug/ml) and Km (50 ug/ml). Finally, the complementation strain was confirmed by PCR and picked for further research. All primer sequences used in this study are listed in Supplementary Table S2.

### Growth curve assay

The bacterial growth assay was carried out according to the instructions (Liu et al., 2019). In brief, *A. citrulli* strains, containing the wild-type (WT) strain, the *oxyR* mutant, and the *oxyR* complementation strain, were all cultured in LB liquid medium overnight at 28°C with shaking at 220 rpm. These strains were then diluted to a final cell density (OD_600_ = 0.01) in 25 ml of fresh LB medium. The diluted cells were cultured at 28°C with shaking at 220 rpm. The bacterial populations were investigated by measuring OD_600_ at 2 h intervals for 24 h. The experiments were performed in triplicate and repeated three times.

### Catalase activity assay

The catalase activity was analyzed using a protocol previously described (Jittawuttipoka et al., 2009). The bacterial strains were cultured in LB broth overnight at 28°C with shaking at 220 rpm. The bacterial centrifugation was adjusted to an OD_600_ = 1.0 with fresh LB broth. The bacterial cells were chilled at 4°C, collected by concentration at 6,000 g for 10 min, and then re-suspended in 50 mmol KH_2_PO_4_. The re-suspended cells were crushed by sonication until the suspension became clear. The cell extracts were separated by centrifuging at 12,000 g for 30 min, and the upper layer liquid containing protein was collected into a new tube. Before and after adding H_2_O_2_ to a final concentration of 10 mm, 100 μl of protein was combined with 1 ml of ddH_2_O, and the optical density of this combination was measured at 240 nm. By using an extinction coefficient of 43.6 M^−1^ cm^−1^ at 240 nm, the catalase activity was determined. Under the assay conditions, one unit of catalase activity was defined as the amount of activity required to degrade 1 μmol of H_2_O_2_ per minute. The experiment was repeated three times with three biological replicates of each treatment.

### Detection of H_2_O_2_ in melon leaves

The H_2_O_2_ was detected by DAB staining as previously reported (Thordal-Christensen et al., 1997; Yu et al., 2016). Briefly, overnight cultures of *A. citrulli* strains were collected by centrifugation and adjusted to a concentration of OD_600_ = 0.3 with sterile double-distilled water, and cells were infiltrated into melon leaves grown for 1 week. The leaf sections (3–5 mm) at 24 h post-inoculation were cut and placed in water with 0.01% Triton-X-100 and DAB at 1 mg/ml, then the leaves were incubated for 8 h at room temperature. Finally, leaves were boiled with 95% ethanol for 10 min and then rinsed with water, and the presence of H_2_O_2_ was visualized as reddish brown colored spots by a light microscope. The experiment was repeated three times with three biological replicates of each treatment.

### H_2_O_2_ sensitivity assay

The diameters of the zones of inhibition for *A. citrulli* WT and derived strains on LB agar plates containing different concentrations of H_2_O_2_ were measured to investigate the roles of *oxyR*, *catB*, and *ahpC* in *A. citrulli* sensitivity to H_2_O_2_. All tested strains were cultured in LB broth at 28°C with 220 rpm shaking until OD_600_ reached 1.0. 100 ml of LB agar medium and 1 ml of cell suspension were well combined, then poured into Petri dishes. An approximately 0.4 cm-diameter sterilized paper disk was put on the middle of each plate following the solidification of these LB agar plates, and 5 μl of various H_2_O_2_ concentrations (1, 5, and 10%) were applied to the disk. After a 24-h incubation at 28°C, the diameters of H_2_O_2_ inhibition zones were measured. Each treatment in this experiment was replicated three times.

### Hypersensitive response assays

To test *A. citrulli* strains’ potential to induce HR, cell suspensions were injected into *Nicotiana benthamiana* leaves. All tested strains were grown in LB and washed with sterile water, then adjusted to OD_600_ = 0.3. Approximately 100 μl of cell suspensions was syringe-infiltrated into the *N. benthamiana* leaves growing at 28°C, and HR was noticed after 24 to 72 h. Each experiment was repeated three times.

### Twitching motility, biofilm formation, and swimming motility assay

The twitching motility of *A. citrulli* strains was assessed as described previously (Bahar et al., 2009), with the following adjustments. *A. citrulli* strains were adjusted to a concentration of approximately 1 × 10^5^ CFU/ml (Dilute 10^3^ fold from OD_600_ = 0.3) with sterilized double-distilled H_2_O. All strains were grown on 1%NA agar plates for 72 h, and twitching motility was observed by Stereo Fluorescence Microscope (Nikon). The characteristic of twitching motility was the formation of a thin and light halo around the colony.

Assays for biofilm formation were carried out in the manner described by Liu et al. (2019). The strains were cultured in LB broth at 28°C and adjusted to OD_600_ = 1.0. The OD_600_ was then determined after 48 h at 28°C by adding 40 μl of the cell suspension to 4 ml of LB broth in a 12-well polystyrene plate. After the cell medium had been decanted, the plate had been dried for 20 min at 80°C. Biofilms were then dyed for 30 min at room temperature with 1% crystal violet. Biofilm production was suggested by a ring of violet precipitate on the plate’s interior wall. After dissolving the biofilm with 5 ml of ethanol, the OD_590_ was measured.

Swimming motility was assayed as described previously (Liu et al., 2019). *A. citrulli* strains were cultured in LB broth at 28°C and adjusted to OD_600_ = 0.3. The middle of 0.3% agar plates received 3 μl of each bacterial cell suspension. After 3 days of incubation at 28°C, the diameters of the swimming halos on the agar plates were measured. Each experiment was run three times with three replicates of each strain tested.

### Electron microscopy

Transmission electron microscopy (TEM) was used to visualize polar flagella of bacteria grown in culture. Specimens for TEM were prepared as previously described (Bahar et al., 2009).

### RNA isolation and quantitative real-time PCR analysis

The tested bacterial strains were cultured at 28°C with shaking at 220 rpm. The cells were collected and adjusted to OD_600_ = 1.0, then harvested by centrifugation at 10,000 × g for 1 min. Total RNA was extracted using the bacterial RNA kit (OMEGA) and DNase-treated RNA with reverse transcription using the HiScript III RT SuperMix reagent Kit with gDNA Wiper (Vazyme). The cDNA was diluted to 50 ng/uL and used for quantitative real-time (qRT)-PCR with ChamQ Universal SYBR qPCR Master Mix (Vazyme) in an ABI PRISM 7500 real-time PCR machine (Applied Biosystems). In this study, the *A. citrulli* 16S ribosomal RNA gene was used as an internal control. qRT-PCR amplification was conducted according to the following program: 95°C for 30 s, followed by 40 cycles of 95°C for 10 s and 60°C for 30 s, and a final melting curve analysis step from 60 to 95°C. Three biological replicates for each gene were used in triplicate during the experiments. We calculated the fold change in gene expression using the comparative 2^-∆∆ct^ method.

### Western blot assay

To determine the protein expression of *pilA*(*Aave_4679*) and *fliC*(*Aave_4400*) in the absence of *oxyR*, respectively, the plasmid (pBBR1-MCS5) carrying the fragment of *pilA* and *fliC* (contain itself native promoter) fusion with a Flag tag was introduced into the *A. citrulli* wild-type and *oxyR* mutants. The bacterial strains were grown in LB broth at 28°C with shaking at 220 rpm. The bacterial concentration was adjusted to an OD_600_ = 1.0 with fresh LB broth. The cells were chilled at 4°C, and harvested by concentration at 6,000 g for 10 min, while cell sediment was collected for intracellular secreted protein assay. Cell sediment was re-suspended in 100uL phosphate-buffered saline (PBS), and then 100 μl Radio Immunoprecipitation Assay (RIPA) lysates was added (1% Triton X-100, 1% deoxycholate, 0.1% sodium dodecyl sulfate (SDS), added at 10 μl per milligram of cells). The cell lysates were heated at 100°C for 10 min. Then, cell lysates were collected by centrifuge with a rate of 12,000 g for 5 min and frozen at −80°C. Cell lysates were resolved by sodium dodecyl sulfate polyacrylamide gel electrophoresis (SDS-PAGE) and transferred to polyvinylidene difluoride membrane (Millipore, Red Bank, NJ, United States) using the semi-dry blot machine (Bio-RAD, CA, United States). After blocking with 5% milk in Tris-buffered saline containing Tween 0.05% (TBST, pH = 7.5) for 1 h at room temperature, the membrane was probed with a monoclonal antibody specific for the Flag tag (1:5000; Abmart, Shanghai, China), followed by detection with an HRP-conjugated anti-rabbit secondary antibody (No. M21002, Abmart, Shanghai, China). Immunoblots were developed using HyGlo HRP ECL Detection kit (MDBio Inc., Qingdao, China) and visualized using an automatic multi-function image analysis system Tanon-6,600 (Tanon, Shanghai, China). As a loading control, a duplicate protein gel was incubated in staining solution with shaking overnight and then incubated in destaining solution with shaking until the bands could be observed clearly.

### Bacterial one-hybrid assay

In the present study, we proved the potential interaction between the transcriptional regulator OxyR and the promoter of the *fliC* and *pilA* using the bacterial one-hybrid reporter system, which consists of two plasmids pTRG and pBXcmT and *E. coli* XL1-Blue MRF’ kan strain (Wang et al., 2018). In particular, the *fliC* promoter region (344 bp) and *pilA* promoter region (333 bp) were cloned into pBXcmT, generating the recombinant vector pBXcmT-*fliC* and pBXcmT-*pilA* (Supplementary Table S1), respectively. Similarly, the coding region of OxyR (966 bp) was cloned into pTRG, creating the final construct pTRG-OxyR. The vectors pBXcmT-P_*fliC*, pBXcmT-P_*pilA,* and pTRG-OxyR were transformed into XL1-Blue MRF’ kan strain, respectively. If the direct physical binding occurs between OxyR and the *fliC* or *pilA* promoter, the transformed *E. coli* strain containing both pBXcmT-P_*fliC* or pBXcmT-P_*pilA* and pTRG-OxyR grows well on the selective medium, which is a minimal medium containing 5 mm 3-amino-1,2,4-triazole, streptomycin at 8 ug/ml, tetracycline at 12.5 ug/ml, chloramphenicol at 34 ug/ml, and Km at 30 ug/ml (Wang et al., 2018). Furthermore, the cotransformant containing pBX-R2031/pTRG-R3133 served as a positive control (Xu et al., 2016; Wang et al., 2018), while the cotransformants containing the empty pTRG and pBXcmT-P_*fliC* or pBXcmT-P_*pilA* were used as a negative control. The cotransformants containing the pTRG-OxyR and empty pBXcmT were used as another negative control in the present study. All cotransformants strains were spotted onto the selective medium plates and placed at 28°C for 3 to 4 days, then photographed.

### Bacterial two-hybrid assay

The bacterial two-hybrid reporter system was applied to examine the potential interaction between OxyR and FliC/PilA. The bacterial two-hybrid reporter system contains three components: plasmids pTRG and pBT and *E. coli* XL1-Blue MRF’ kan strain. In this study, the coding region of FliC (1,479 bp) and PilA (507 bp) was cloned into pBT, generating the recombinant vector pBT-FliC and pBT-PilA (Supplementary Table S1), respectively. Similarly, the coding region of OxyR (966 bp) was cloned into pTRG, creating the final construct pTRG-OxyR. The vectors pBT-FliC, pBT-PilA, and pTRG-OxyR were transformed into XL1-Blue MRF’ kan strain, respectively. If the direct protein–protein interaction occurs between OxyR and FliC or PilA, the transformed *E. coli* strain containing both pBT-FliC or pBT-PilA and pTRG-OxyR grows well on the selective medium, which is similar to the components of bacterial one-hybrid system. Furthermore, the cotransformant containing pBT-GacS/pTRG-GacS served as a positive control, while the cotransformants containing the empty pTRG and pBT-FliC or pBT-PilA worked as a negative control. The cotransformants containing the pTRG-OxyR and empty pBT worked as another negative control in the present study. All cotransformants strains were spotted onto the selective medium and grown at 28°C for 3 to 4 days, then photographed.

### Virulence and colonization of *Acidovorax citrulli* assay

In this study, two inoculation methods, including injection of melon seedling cotyledons and seed-to seedling transmission assay, were applied to examine the virulence of *A. citrulli* strains. For injection of melon seedling cotyledons, overnight cultures of *A. citrulli* strains were collected by centrifugation and adjusted to a concentration of about 1 × 10^3^ CFU/ml with sterile double-distilled water. Each strain was inoculated onto 10 melons (cv. Huanghou) cotyledons (on 1-week-old seedlings). These inoculated plants were then incubated at 28°C and checked for disease symptoms at 5 days post-inoculation. For seed-to seedling transmission assay, 25 melon seeds (cv. Huanghou) were soaked in cell suspensions (approximately 1 × 10^6^ CFU/ml) of each strain for 2 h before air-dried at room temperature. Five seeds were planted per cup (Wuhao) and incubated at 28°C with 100% RH. After 7 days, the seedlings were observed for BFB symptoms. Meanwhile, 25 seedlings inoculated with double-distilled water were used as negative controls. This experiment was carried out three times.

The seedling colonization of *A. citrulli* wild-type and its derived mutant strains was determined by infiltrating melon cotyledons and seed. The bacterial cells with 1 × 10^3^ CFU/ml were injected into 15 melon cotyledons (cv. Huanghou) using a sterile syringe. Sterilized water served as a negative control. The inoculated melon seedlings were incubated for 0, 24, 48, 72, and 96 h in a growth chamber with 100% RH at 28°C. The inoculated melon cotyledons were then crushed and put into a 1.5-mL centrifuge tube with 100 μl of sterilized water. The homogenate was diluted 10-, 100-, and 1,000-fold, and 100 μl of the homogenate was spread onto LB plates with appropriate antibiotics. After incubating at 28°C for 24 to 96 h, colonies were counted. According to earlier reports (Tian et al., 2014), the surface of the seed was disinfected with 70% ethanol for 5 min before inoculation. The front end of the seed was opened and each bacterial cell suspension (approximately 1 × 10^3^ CFU/ml) was implanted into 15 melon seeds (Huanghou). Additionally, 15 melon seeds treated with sterilized water served as a negative control. All melon seeds were incubated for 24, 48, 72, and 96 h at 28°C with 100% RH on moist blotter papers. All melon seeds were milled before transferring to a 1.5-ml centrifuge tube with 100 μl of sterilized water. Then, the seed homogenate was diluted 10-, 100-, and 1,000-fold, and 100 μl of the homogenate was spread onto LB agar plates with Rif (100 ug/ml). After incubation at 28°C for 24 to 96 h, colonies were counted. These experiments were repeated three times.

## Results

### OxyR is present in the *Acidovorax citrulli* AAC00-1 genome

A gene encoding OxyR protein was identified in the genome of *A. citrulli* based on comparisons of amino acid sequences from other plant pathogenic bacteria. The *oxyR* open reading frame (ORF) was 966 bp in length and located in the *A. citrulli* AAC00-1 genome at nucleotide position 651,313 to 652,278 (GenBank accession number NC_008752). The NCBI BLAST was used to perform BLASTP sequence homology analysis. Multiple sequence alignment shows that the amino acid sequence of OxyR from *A. citrulli* has a high identity among the tested bacteria including *E. coli*, *Pseudomonas aeruginosa*, *P. syringae* pv. *tomato,* and *Xanthomonas. oryzae* pv. *oryzae* with 40, 49, 48, and 43% amino acid sequence identities, respectively. (Supplementary Figure S1). Subsequently, we constructed *A. citrulli oxyR* mutant (*Ac*Δ*oxyR*) and complementation strain *Ac*Δ*oxyR* (pBBR-OxyR) by homologous recombination. We monitored bacterial growth in Luria-Bertani (LB) liquid media. The growth rate of *Ac*Δ*oxyR* was similar to that of WT and *Ac*Δ*oxyR* (pBBR-OxyR) strains (Supplementary Figure S2).

### *Acidovorax citrulli* OxyR is required for catalase activity and response to H_2_O_2_

**Figure 1:**
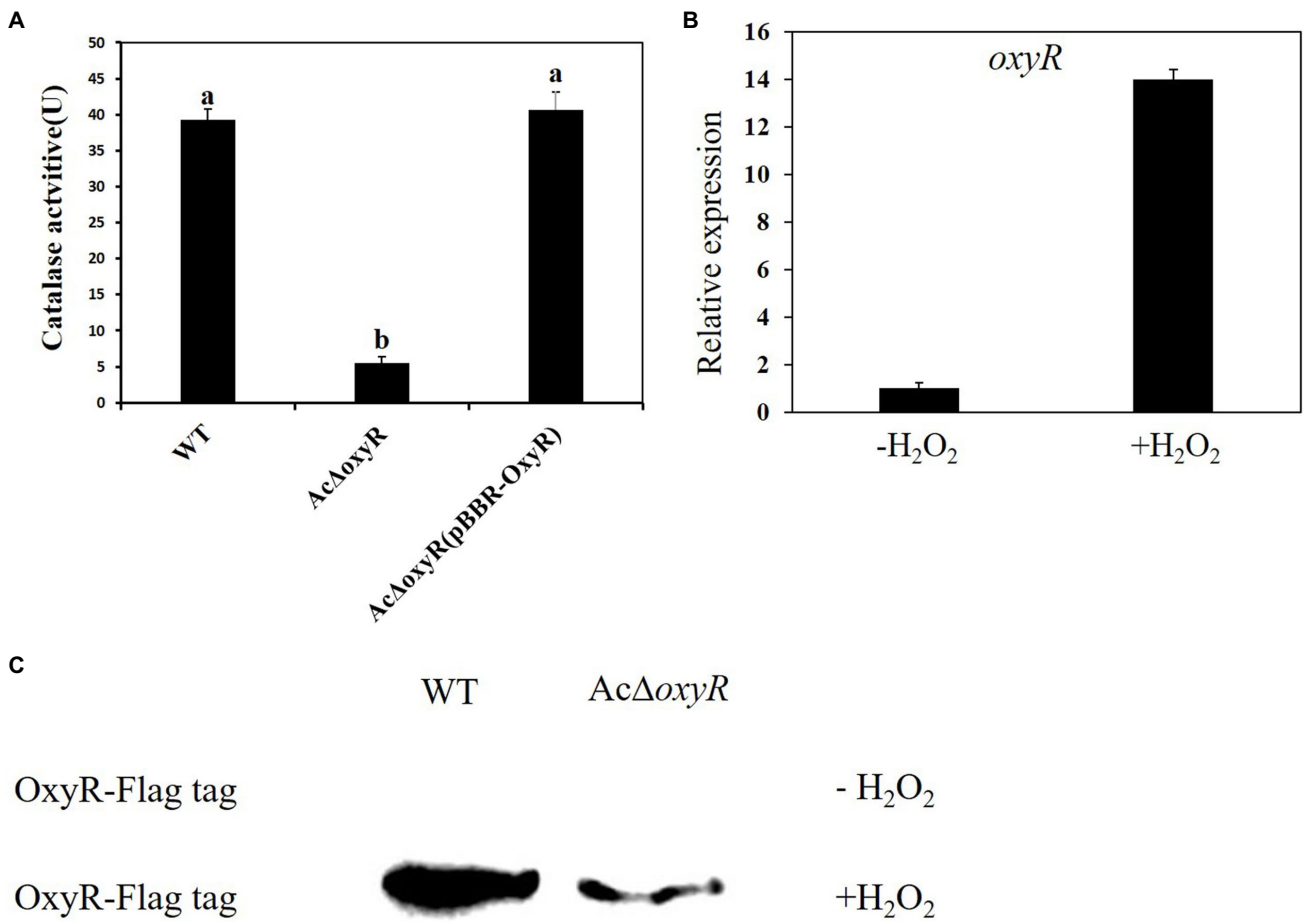
Assays for catalase activity and expression of *oxyR* of *A. citrulli* strains in response to H_2_O_2_. **(A)** Catalase activity assays. Catalase activities associated with cell extracts were assessed by spectrophotometric assay. One unit of catalase activity was defined as the amount of activity required to decompose 1 μmol of H_2_O_2_ per minute under the assay conditions. Different lowercase letters indicate a significant difference between treatments. Statistically significant differences were determined by the one-way ANOVA of variance and *p* < 0.05. **(B)** Expression of the *oxyR* in *A. citrulli* WT grown in LB medium with or without 1 mm H_2_O_2_ and determined by qRT-PCR. Error bars indicate standard deviations. Statistically significant differences were determined by the one-way ANOVA of variance and *p* < 0.05. **(C)** Abundance of OxyR-Flag in the WT and *Ac*Δ*oxyR* grown in LB medium with or without 1 mm H_2_O_2_ and determined by western blot analysis. Experiments were repeated three times with similar results.

To determine the roles of the *oxyR* gene mediating H_2_O_2_ detoxification, we measured the catalase activities of the *A. citrulli* WT, *Ac*Δ*oxyR,* and *Ac*Δ*oxyR* (pBBR-OxyR; Figure 1A). The catalase activity of *Ac*Δ*oxyR* was significantly reduced as compared with the WT, while the complementation strain *Ac*Δ*oxyR* (pBBR-OxyR) restored to the WT level. In addition, we examined the expression level of *oxyR* in WT under H_2_O_2_ stress. Our results demonstrated that the expression of *oxyR* was significantly increased after 30 min of treatment with 1 mm H_2_O_2_ (Figure 1B). In addition, the western blot displayed that the expression of OxyR was not detected in both WT and *Ac*Δ*oxyR* without H_2_O_2_ treatment (Figure 1C). However, when supplement with exogenous H_2_O_2_, OxyR expression of WT was significantly increased than *Ac*Δ*oxyR* (Figure 1C). These results suggested that OxyR plays an important role in catalase production and response to oxidative stress in *A. citrulli.*

### *Ac*Δ*oxyR* elicit H_2_O_2_ production in melon

We detected the production of H_2_O_2_ in melon leaves at 24 h post-inoculation of WT, *Ac*Δ*oxyR,* and *Ac*Δ*oxyR* (pBBR-OxyR) by using 3, 3′-diaminobenzidine (DAB) staining. The red spots formed by DAB staining in all areas of the melon leaves inoculated with bacteria represented the accumulation of H_2_O_2_, while the ddH_2_O control had no accumulation of H_2_O_2_ (Supplementary Figure S3). As a result, these findings suggest that WT, *Ac*Δ*oxyR,* and *Ac*Δ*oxyR* (pBBR-OxyR) elicit H_2_O_2_ production in melon at the early stages of infection.

### Role of *Acidovorax citrulli* OxyR in H_2_O_2_ tolerance

**Figure 2:**
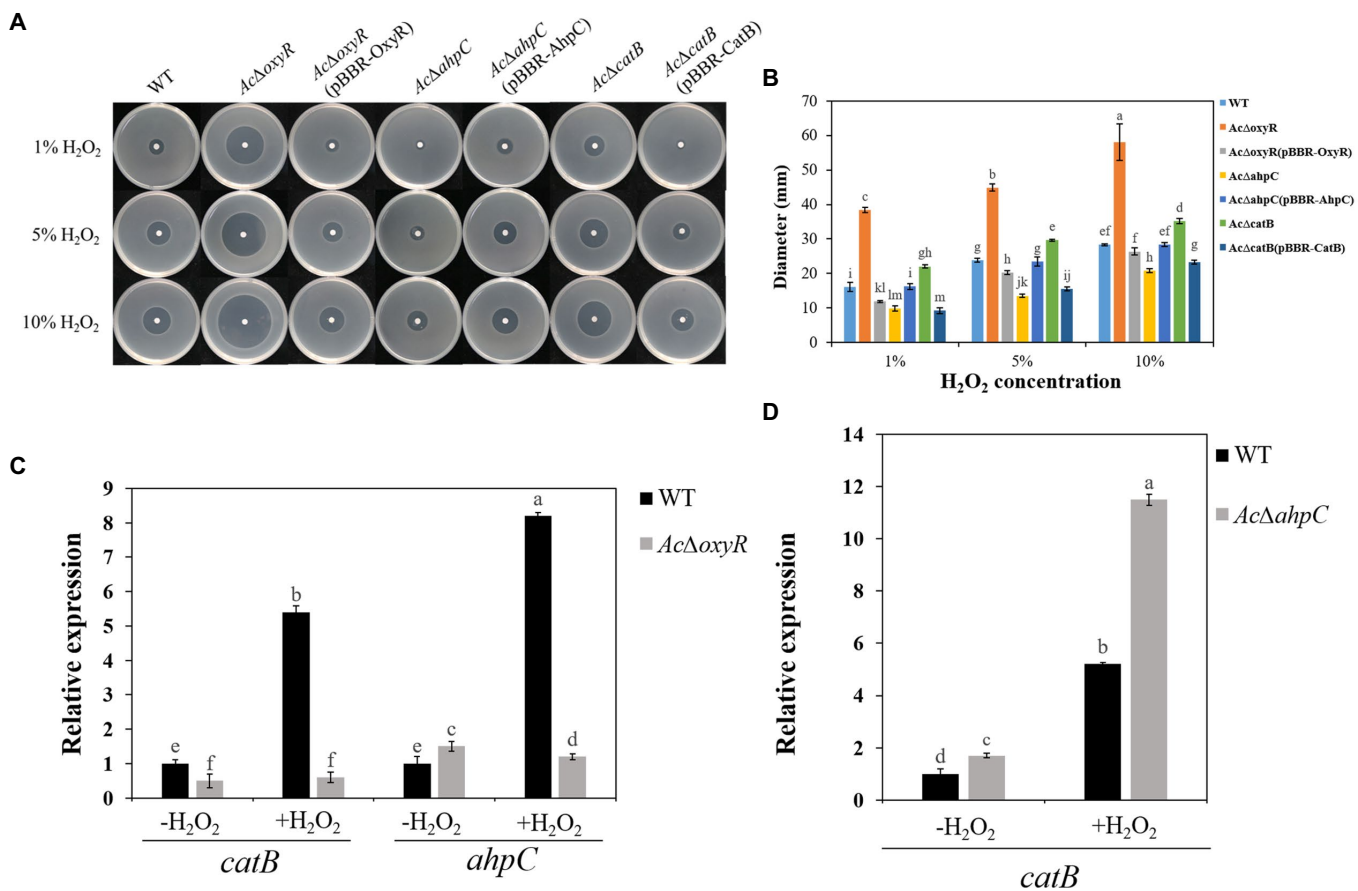
Sensitivity of WT, *Ac*Δ*oxyR*, *Ac*Δ*oxyR*(pBBR-OxyR), *Ac*Δ*ahpC*, *Ac*Δ*ahpC*(pBBR-AhpC), *Ac*Δ*catB*, *Ac*Δ*catB*(pBBR-CatB), and WT(pBBR-CatB) strains to H_2_O_2_. **(A)** Three microliters of different concentrations (1, 5, and 10%) of H_2_O_2_ were dropped in the center of the plates. The H_2_O_2_ inhibition zones were observed and measured after incubation at 28°C for 24 h. **(B)** The diameter of the zone of bacterial growth inhibition. **(C)** Expression of *catB* and *ahpC* in *A. citrulli* WT and *Ac*Δ*oxyR* grown in LB medium with or without 1 mm H_2_O_2_ and determined by qRT-PCR. **(D)** Expression of *catB* in *A. citrulli* WT and *Ac*Δ*ahpC* grown in LB medium with or without 1 mm H_2_O_2_ and determined by qRT-PCR. Experiments were performed in triplicate and were repeated three times with similar results. Data represent the means of three replicates ± standard deviations (error bars). Different lowercase letters indicate a significant difference between treatments. Statistically significant differences were determined by the one-way ANOVA of variance and *p* < 0.05.

To investigate whether *A. citrulli* OxyR plays an important role in H_2_O_2_ response, we examined the WT, *Ac*Δ*oxyR*, *Ac*Δ*ahpC*, *Ac*Δ*catB*, and their complementation strains *Ac*Δ*oxyR* (pBBR-OxyR), *Ac*Δ*ahpC* (pBBR-AhpC), and *Ac*Δ*catB* (pBBR-CatB) to H_2_O_2_ sensitivity based on the diameter of the inhibition zone (Figure 2A). Both the *Ac*Δ*oxyR* and *Ac*Δ*catB* mutants showed significantly increased sensitivity, while the *Ac*Δ*ahpC* mutant showed significantly decreased sensitivity of H_2_O_2_ as compared with the WT(Figure 2B). The H_2_O_2_ sensitivity of all complementation strains *Ac*Δ*oxyR* (pBBR- OxyR), *Ac*Δ*ahpC* (pBBR- AhpC), and *Ac*Δ*catB* (pBBR- CatB) was similar with WT (Figure 2B). Furthermore, qRT-PCR assay showed that *catB* and *ahpC* expression levels were significantly down-regulated in *Ac*Δ*oxyR* under H_2_O_2_ stress environment (Figure 2C). In addition, the expression level of *catB* was significantly up-regulated in *Ac*Δ*ahpC* under H_2_O_2_ stress environment (Figure 2D). These results suggested that OxyR played a role in protecting *A. citrulli* cells in H_2_O_2_ stress.

### OxyR is involved in *Acidovorax citrulli* HR induction and positively regulates expression of genes related to T3SS

**Figure 3:**
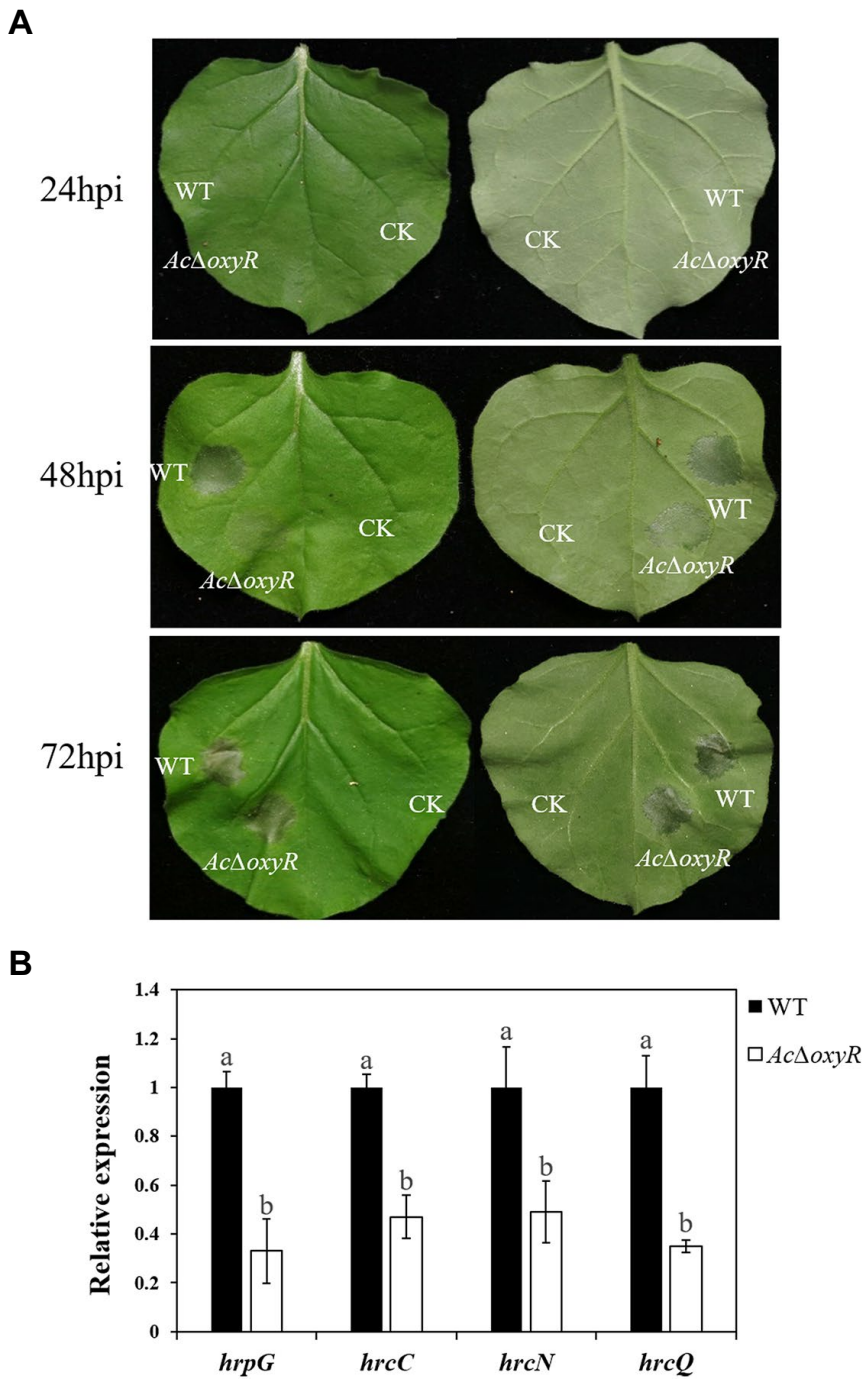
Effects of *oxyR* on *Nicotiana benthamiana* hypersensitive response (HR) induction by *Acidovorax citrulli* strains. **(A)** Tobacco HR induction. Approximately 100 μl of bacterial suspension (optical density at 600 nm = 0.3) was infiltrated at each inoculation site on a tobacco leaf and the plant was incubated at 28°C. *N. benthamiana* was grown under greenhouse conditions at 24°C and observed for HR at 24 to 72 h post-infiltration(hpi). CK: double-distilled H_2_O, WT: wild-type xjl12, *Ac*Δ*oxyR*: *oxyR* gene deletion mutant. **(B)** Expression level of *hrpG*, *hrcN*, *hrcC,* and *hrcQ* genes in WT and *Ac*Δ*oxyR* strains by qRT-PCR. Experiments were conducted in triplicate and repeated three times with similar results. Data shown represent the means of three replicates ± standard deviations. Error bars indicate standard deviations. Different lowercase letters indicate a significant difference between strains. Statistically significant differences were determined by the one-way ANOVA of variance and *p* < 0.05.

To determine whether OxyR contributes to the *A. citrulli* type III secretion system (T3SS), we examined the HR induction of WT and *Ac*Δ*oxyR* on *Nicotiana benthamiana*. At 24 h post-infiltration (hpi), both the WT and *Ac*Δ*oxyR* did not induce HR. HR induction on *N. benthamiana* by *Ac*Δ*oxyR* was delayed than that induced by the WT at 48 hpi, while *Ac*Δ*oxyR* was no different than WT at 72 hpi (Figure 3A). Furthermore, qRT-PCR was used to confirm the regulatory effect on the T3SS-related genes. We identified that T3SS genes including *hrpG*, *hrcC*, *hrcN,* and *hrcQ* were significantly down-regulated in *Ac*Δ*oxyR* relative to the WT strain (Figure 3B). These results indicate that OxyR positively regulates the expression of genes related to T3SS.

### Role of OxyR in twitching motility, biofilm production and swimming motility of *Acidovorax citrulli*

**Figure 4:**
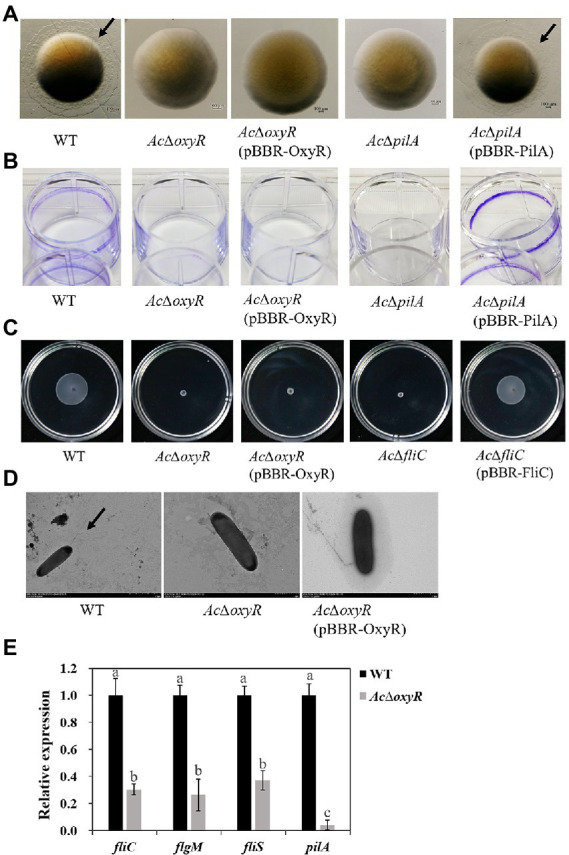
Role of *oxyR* in twitching motility, biofilm production, and swimming motility of *A. citrulli*. **(A)** Twitching motility of *A. citrulli* strains including *A. citrulli* wild-type (WT), *Ac*Δ*oxyR*, *Ac*Δ*pilA,* and complementation strains *Ac*Δ*oxyR* (pBBR-OxyR) and *Ac*Δ*pilA* (pBBR-PilA). Strains were painted on nutrient agar plates containing 1.0% agar. The colonies were photographed and observed using a stereoscope after 72 h. Black lines indicate the twitching halo. **(B)** Biofilm formation of *A. citrulli* strains including *A. citrulli* wild-type (WT), *Ac*Δ*oxyR*, *Ac*Δ*pilA,* and complementation strains *Ac*Δ*oxyR* (pBBR-OxyR) and *Ac*Δ*pilA* (pBBR-PilA). Biofilm production is indicated by a ring of dark precipitate on the inner wall of culture plate wells. **(C)** Swimming motility of *A. citrulli* strains including *A. citrulli* wild-type (WT), *Ac*Δ*oxyR*, *Ac*Δ*fliC,* and complementation strains *Ac*Δ*oxyR* (pBBR-OxyR) and *Ac*Δ*fliC* (pBBR-FliC) on 0.3% agar plates at 28°C for 3 days. **(D)** Transmission electron microscope verification of presence or absence of polar flagella. Full-length flagella (arrows) can be seen in WT strain, while none of flagella were seen in *Ac*Δ*oxyR* and *Ac*Δ*oxyR* (pBBR-OxyR). **(E)** Expression level of flagella-related genes (*fliC*, *fliS* and *flgM*) and *pilA* between *A. citrulli* WT and *Ac*Δ*oxyR* were determined by qRT-PCR. Different lowercase letters indicate a significant difference between treatments. Statistically significant differences were determined by the one-way ANOVA of variance and *p* < 0.05.

Previous studies have confirmed that motility and biofilm play a key role in the virulence of *A. citrulli*. Previous studies have reported that *pilA* is required for biofilm formation for scoliosis, while *fliC* is required for swimming motility in *A. citrulli* M6 strain (Bahar et al., 2009, 2011). We investigated the role of OxyR in *A. citrulli* twitching motility, biofilm production, and swimming motility. Transparent halos around colonies formed by *A. citrulli* strains by twitching motility on NA plates were observed after 72 h at 28°C. In NA, *Ac*Δ*oxyR* exhibited a phenotype similar to that of *Ac*Δ*pilA* (i.e., no twitching-typical haloes were formed around the colonies; Figure 4A). In addition, the biofilm production of both *Ac*Δ*oxyR* and *Ac*Δ*pilA* was significantly decreased than the WT and *Ac*Δ*pilA* (pBBR- ilA; Figure 4B; Supplementary Figure S4). The swimming ability of *Ac*Δ*oxyR* and *Ac*Δ*fliC* was completely lost(Figure 4C), and *Ac*Δ*oxyR* did not produce polar fagella (Figure 4D). However, the complementation strain *Ac*Δ*oxyR* (pBBR-OxyR) formed the same phenotype as *oxyR* mutants *Ac*Δ*oxyR* in twitching motility, biofilm production and swimming motility. Furthermore, the qRT-PCR revealed that the expression levels of *fliC, pilA, fliS,* and *flgM* were down-regulated in the *Ac*Δ*oxyR* as compared with WT (Figure 4E). These results indicated that *oxyR* gene plays a role in twitching motility, swimming motility, and biofilm production in *A. citrulli*.

### OxyR positively regulates the expression of FliC and PilA

**Figure 5:**
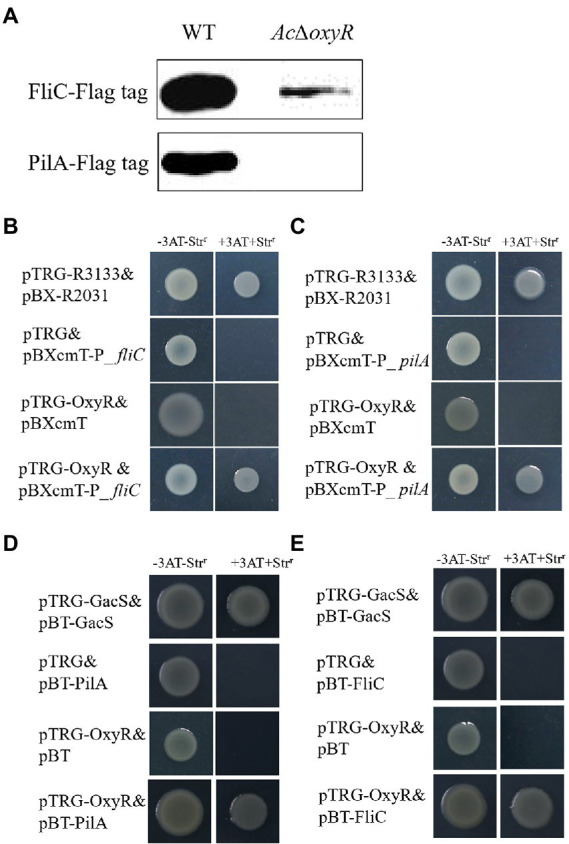
Expression of PilA and FliC directly affected by OxyR. **(A)** Abundance of FliC-Flag and PilA-Flag in the WT and *Ac*Δ*oxyR* were analyzed by western blot. **(B)** The direct physical interaction between OxyR and the *fliC* promoter region was detected in *E. coli*. **(C)** The direct physical interaction between OxyR and the *pilA* promoter region was detected in *E. coli*. **(D)** OxyR and PilA interaction verified by bacterial two-hybrid. OxyR was cloned into vector pTRG, and PilA were cloned into vector pBT, respectively. **(E)** OxyR and FliC interaction verified by bacterial two-hybrid. OxyR was cloned into vector pTRG, and FliC was cloned into vector pBT, respectively. pTRG-R3133& pBX-R2031: co-transformant containing pBX-R2031 and pTRG-R3133, serves as a positive control; pTRG-OxyR& pBXcmT: co-tranformant containing pTRG-OxyR and empty pBXcmT; pTRG& pBXcmT-P_ *fliC*: co-tranformant containing pBXcmT-P_*fliC* and empty pTRG; pTRG& pBXcmT-P_ *pilA*: co-tranformant containing pBXcmT-P_ *pilA* and empty pTRG; pTRG-OxyR& pBXcmT-P_*fliC*: co-transformant possessing both pTRG-OxyR and pBXcmT-P_*fliC*; pTRG-OxyR& pBXcmT-P_ *pilA*: co-transformant possessing both pTRG-OxyR and pBXcmT-P_ *pilA*; pTRG-GacS& pBT-GacS: co-transformant containing pTRG-GacS and pBT-GacS, serves as a positive control; pTRG-OxyR& pBT: co-tranformant containing pTRG-OxyR and empty pBT; pTRG & pBT-PilA: co-tranformant containing pBT-PilA and empty pTRG; pTRG& pBT-FliC: co-tranformant containing pBT-FliC and empty pTRG; pTRG-OxyR & pBT-PilA: co-transformant possessing both pTRG-OxyR and pBT-PilA; pTRG-OxyR & pBT-FliC: co-transformant possessing both pTRG-OxyR and pBT-FliC. -3AT-Str^r^: no selective LB medium plate; +3AT + Str^r^: M9-based selective medium plate.

To determine the relationship of OxyR to PilA and FliC, we performed the western blot, bacterial one-hybrid, and bacterial two-hybrid assays. The western blot assay indicated that the expression of PilA and FliC was down-regulated in *Ac*Δ*oxyR* (Figure 5A). In addition, a newly-developed bacterial one-hybrid system (Wang et al., 2018) was carried out to test the potential direct interaction between OxyR and the *fliC* and *pilA* promoter (P_*fliC* and P_*pilA*). We observed that the growth of *E. coli* strain containing both OxyR and P_*fliC* was similar to positive control on the selective medium, whereas the negative controls failed to grow (Figure 5B). The same observation occurred on the test *E. coli* strain containing both OxyR and P_*pilA* (Figure 5C). These results indicated that direct binding of OxyR on P_*fliC* and P_*pilA* occurred under the test conditions. To verify whether OxyR interacts with PilA, the bacterial two-hybrid system was performed. Bacterial two-hybrid assays displayed that the growth of *E. coli* strain containing both OxyR and PilA was similar to positive control on the selective medium (Figure 5D). The same results were observed on selective media for *E. coli* strains containing OxyR and FliC (Figure 5E). These results indicated that OxyR interacts with PilA and FliC.

### Deletion of OxyR in *Acidovorax citrulli* displayed decreased virulence and bacterial growth on melon

**Figure 6:**
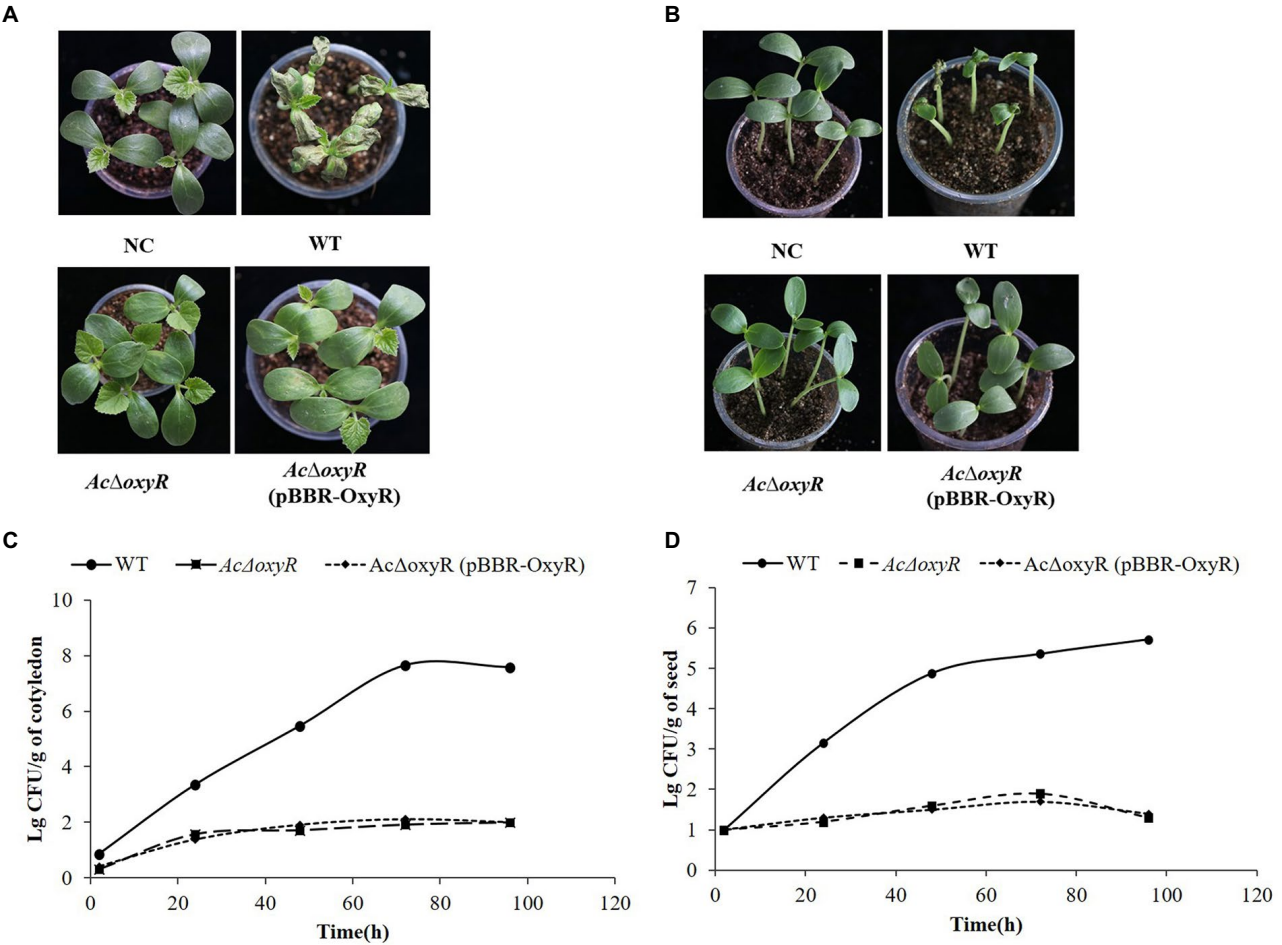
Role of *oxyR* in *Acidovorax citrulli* virulence and bacterial growth on melon. **(A)** Melon seedling cotyledons inoculated by injection with *A. citrulli* wild-type (WT), *Ac*Δ*oxyR*, and *Ac*Δ*oxyR* (pBBR- OxyR; ~1 × 10^3^ CFU/ml) strains, and double-distilled H_2_O (ddH_2_O) as a negative control (NC). Seedlings were observed for bacterial fruit blotch symptoms at 5 days post-inoculation(dpi). **(B)** Melon seeds were inoculated by soaking in bacterial cell suspensions (~1 × 10^6^ CFU/ml) of *A. citrulli* WT, *Ac*Δ*oxyR*, and *Ac*Δ*oxyR* (pBBR-OxyR). BFB symptoms were observed 7 days after planting. **(C)** Bacterial suspensions (~1 × 10^3^ CFU/ml) of each strain were injected into cotyledons of melon seedlings and populations were quantified at 0, 24, 48, 72, and 96 h postinoculation. **(D)** Melon seed were inoculated with *A. citrulli* strains (~1 × 10^3^ CFU/ml) planted under conditions conducive for bacterial fruit blotch development and bacterial populations on emerging seedling tissues were quantified at 0, 24, 48, 72, and 96 h after planting. Experiments were repeated three times with similar results.

To investigate the effect of *oxyR* in virulence of *A. citrulli* on melon, WT, *Ac*Δ*oxyR,* and complementation strain *Ac*Δ*oxyR* (pBBR-OxyR) infiltrated into cotyledons of melon seedlings. In both two virulence assays, *Ac*Δ*oxyR* did not induce BFB symptoms on melon cotyledons (Figures 6A,B). The colonization ability of *A. citrulli* WT, *Ac*Δ*oxyR,* and *Ac*Δ*oxyR* (pBBR-OxyR) strains on melon seedlings was determined. At 0, 24, 48, 72, and 96 hpi, bacterial populations in seedling tissues were evalued. The average cell populations of *A. citrulli* WT, *Ac*Δ*oxyR,* and *Ac*Δ*oxyR* (pBBR-OxyR) strains were approximately 2.95 × 10^5^,50, and 80 CFU/g, respectively, by 48 hpi. By 96 hpi, the mean populations of WT, *Ac*Δ*oxyR,* and *Ac*Δ*oxyR* (pBBR-OxyR) strains were approximately 2.57 × 10^7^, 95, and 100 CFU/g, respectively (Figure 6C). In melon cotyledons, the population growth of *Ac*Δ*oxyR* was noticeably decreased than WT strain. Additionally, by monitoring the bacterial populations on artificially inoculated seeds throughout the first 96 h of seed germination, we assessed the effect of *oxyR* in *A. citrulli* colonization of melon seeds. At 0, 24, 48, 72, and 96 h after planting, the bacterial populations on the emerging tissues of melon seedlings were enumerated. The average cell populations of WT, *Ac*Δ*oxyR,* and *Ac*Δ*oxyR* (pBBR-OxyR) were approximately 7.59 × 10^4^, 40, and 32 CFU/g, respectively, at 48 h after planting (Figure 6D). The average populations of WT, *Ac*Δ*oxyR,* and *Ac*Δ*oxyR* (pBBR-OxyR) were approximately 5.25 × 10^5^, 20, and 25 CFU/g, respectively, by 96 h after planting (Figure 6D). Compared to the WT strain, *Ac*Δ*oxyR* had a much lower capacity to colonize germinating melon seedlings (*p* < 0.05). These results demonstrated that OxyR was required for the full virulence of *A. citrulli* on melon.

## Discussion

OxyR plays an important role in oxidative stress, alginate biosynthesis, T3SS-related gene expression, and virulence of many plant-pathogenic bacteria, including *Xanthomonas oryzae* pv. *oryzae* (Yu et al., 2016), *Pseudomonas syringae* pv. *tomato* (Ishiga and Ichinose, 2016), and *Xanthomonas campestris* pv. *phaseoli* (Loprasert et al., 2000). However, to date, there have been no reports regarding the role of OxyR in *Acidovorax citrulli*. In this study, we functionally characterized the role of *oxyR* in *A. citrulli* wild-type strain xjl12 by constructing an *oxyR* mutant (*Ac*Δ*oxyR*) and a corresponding *oxyR*-complementation strain *Ac*Δ*oxyR* (pBBR-OxyR). In *Bacteroides fragile* (Sund et al., 2007), and *Haemophilus parasuis* (Wen et al., 2018), Δ*oxyR* exhibited markedly impaired growth compared to the WT strain. Conversely, the deletion of *oxyR* did not affect the bacterial growth *in vitro* in *A. citrulli* (Supplementary Figure S2) and *P. syringae* (Ishiga and Ichinose, 2016).

Reactive oxygen species (ROS) can damage all cellular components, including protein, DNA and membrane lipid (Imlay and Linn, 1988; Fridovich, 1997). Therefore, pathogenic bacteria successfully infect plant tissues in part by depending on their abilities to resist ROS, including H_2_O_2_ (Cabiscol et al., 2000). OxyR was identified as the primary H_2_O_2_ sensor responsible for H_2_O_2_ resistance (Jo et al., 2015). Previously reported that bacterial CAT was involved in the H_2_O_2_-degradation pathway and increased tolerance to oxidative stress (Loprasert et al., 1996). In this study, the *Ac*Δ*oxyR* was significantly reduced the CAT activity as compared with the WT. Furthermore, the transcription and translation levels of *oxyR* were induced by exogenous H_2_O_2_. These results indicated that *oxyR* was activated by H_2_O_2_ and then play a key role in the H_2_O_2_ degradation pathway. Moreover, the accumulation of H_2_O_2_ was observed in melon leaves infected by *A. citrulli* WT, *Ac*Δ*oxyR*, and *Ac*Δ*oxyR* (pBBR-OxyR) strains, suggesting that the ability of *A. citrulli* strains to degrade H_2_O_2_ is a key determinant of host infection by *A. citrulli* strains.

OxyR is a transcription factor regulates several genes involved in anti-oxidative stress, such as *katA* and *katB* (encoding catalases A and B) and *ahpC* and *ahpF* (encoding an alkyl hydroperoxide reductase) (Ochsner et al., 2000; Hishinuma et al., 2006). To investigate the sensitivity to H_2_O_2_ of *oxyR* and its regulatory gene, *catB* and *ahpC* that encode a catalase and an alkyl hydroperoxide reductase, respectively, were deleted. In this study, *oxyR* and *catB* mutants were significantly reduced the tolerance to H_2_O_2_. In contrast, *ahpC* mutant was significantly increased the tolerance to H_2_O_2_. These results showed that deletion of *ahpC* may be due to increased expression of other oxidative stress-related genes thus triggering compensation mechanism. Using a qRT-PCR assay, we demonstrated that *catB* and *ahpC* were down-regulated in *Ac*Δ*oxyR*. In addition, *catB* was significantly up-regulated in *Ac*Δ*ahpC*. These results represented that OxyR might be on the top of the antioxidant stress regulatory system by controlling the expression of other oxidative stress-related genes. In the future, we would further analyze the antioxidant stress pathway of *oxyR* in *A. citrulli*.

Deletion of *oxyR* displayed reduced virulence and the ability to colonize melon seedlings. *Ac*Δ*oxyR* may influence other virulence factors, e.g., T3SS, biofilm, and swimming motility. The T3SS is major pathogenicity and virulence factors in *A. citrulli* (Johnson et al., 2009; Bahar and Burdman, 2010). In *Pst*DC3000, the expression of *hrpL* and *corR* were down-regulated in *oxyR* mutant (Ishiga and Ichinose, 2016). In this study, the T3SS-related genes including *hrpG*, *hrcN*, *hrcC,* and *hrcQ* were significantly down-regulated in *Ac*Δ*oxyR* relative to the WT strain. This result displayed that OxyR positively regulated the expression of T3SS-related genes that contribute to virulence.

In previous reports, *pilA* was required for twitching motility, and biofilm formation of *Acidovorax avenae* subsp. c*itrulli* (Bahar et al., 2009). In *A. citrulli, fliC* encodes the flagellin subunit and plays an important role in swimming motility (Bahar et al., 2011). The formation of biofilm and swimming motility was significantly inhibited in the *oxyR* mutant (Chung et al., 2016). In this study, *Ac*Δ*oxyR* and *Ac*Δ*pilA* were deficient in twitching motility and biofilm formation. Furthermore, *Ac*Δ*oxyR* was completely lost the ability of swimming motility and it is similar to the deletion of *fliC.* The complementation strain *Ac*Δ*oxyR* (pBBR-OxyR) formed the same phenotype as *Ac*Δ*oxyR* in twitching motility, biofilm produce, and swimming motility. Similar results existed in the virulence assay, *Ac*Δ*oxyR* and complementation strain *Ac*Δ*oxyR* (pBBR-OxyR) both reduced virulence and bacterial growth in *planta*. These results indicated that deletion of *oxyR* in *A. citrulli* may be affected the function of adjacent genes. Therefore, complementation strain *Ac*Δ*oxyR* (pBBR-OxyR) cannot restore the function of adjacent genes, thus losing these phenotypes of twitching motility, biofilm production, and swimming motility. The speculation will be further studied in the future. Moreover, complementation strain *Ac*Δ*oxyR* (pBBR-OxyR) could restore the tolerance to H_2_O_2_, but not restore the phenotypes of biofilm, swimming, and twitching motility, thus failing to restore the virulence of *A. citrulli*. This result indicates that OxyR affects the virulence in *A. citrulli* by regulating multiple virulence traits.

To clarify whether OxyR directly regulates motility and biofilm and thus affects the virulence of *A. citrulli*, we demonstrated the interaction between *oxyR* and *pilA* and *fliC* by bacterial one-hybrid system and bacterial two-hybrid system. We demonstrated that the PilA and FliC proteins were significantly reduced in *Ac*Δ*oxyR*, indicated that OxyR positively affects *pilA* and *fliC*. In addition, bacterial one-hybrid system assay indicated the direct interaction between OxyR and the *pilA* and *fliC* promoter. Interestingly, the interaction between OxyR, PilA, and FliC was proved using bacterial two-hybrid system assay. In this study, we demonstrated that direct binding of OxyR on *pilA* and *fliC* promoter and protein–protein interactions occurred under the test conditions. These results showed that OxyR was directly affected *pilA* and *fliC* expression, thus affecting twitching motility, biofilm, and swimming motility of *A. citrulli*.

**Figure 7:**
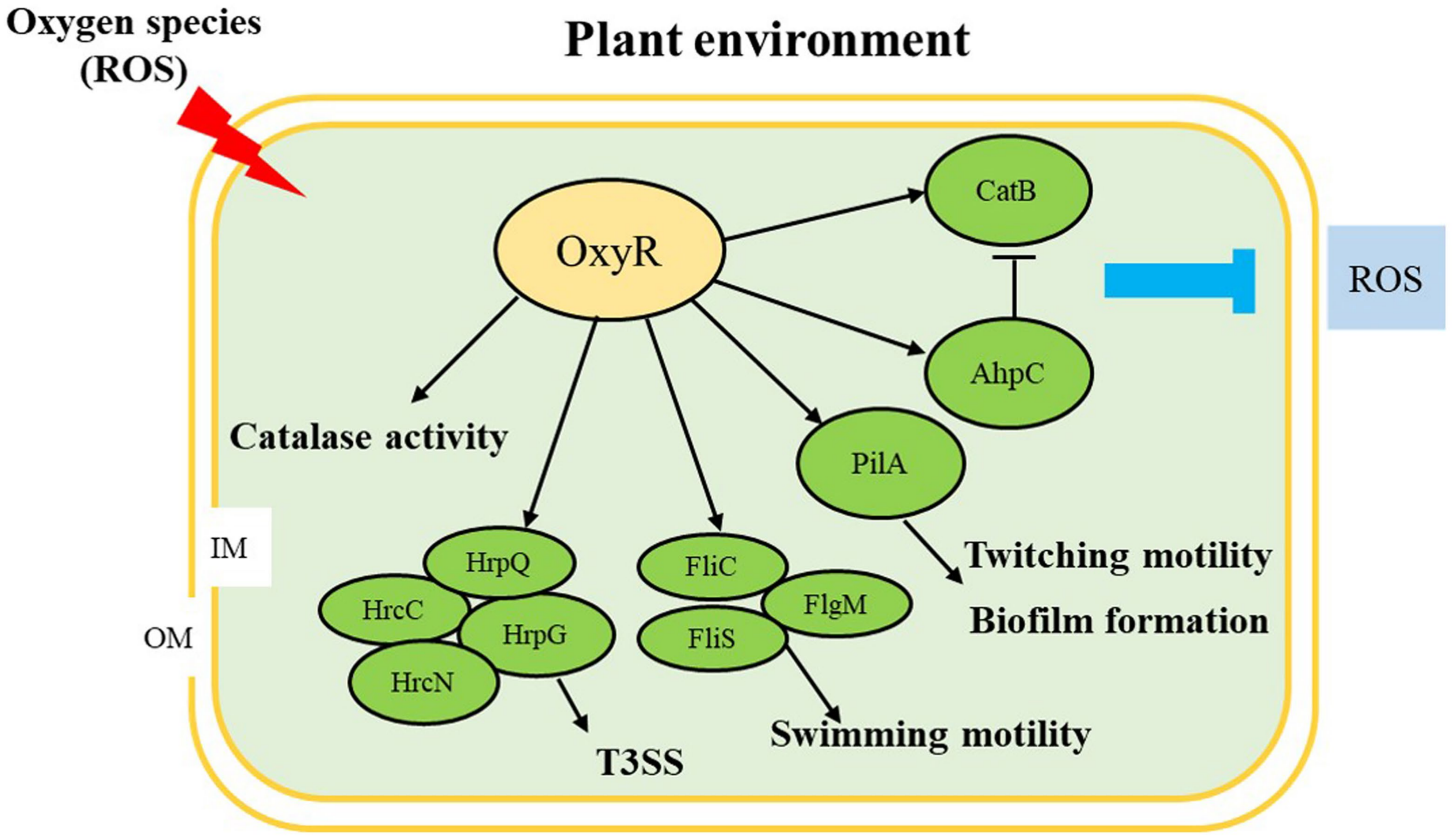
Proposed model illustrating the global effect of OxyR in *Acidovorax citrulli*. ↓, positive regulation; ⊥, negative regulation; OM: outer membrane; IM: inner membrane; ROS: reactive oxygen species; T3SS: type III secretion system. Regulatory steps in the model are mainly at the transcriptional level.

In summary, we observed that OxyR is involved in the regulation of many virulence factors in *A. citrulli*, including oxidative stress response, CAT activity, T3SS, swimming motility, twitching motility, and biofilm formation (Figure 7). We also demonstrated that OxyR directly binds *fliC* and *pilA* promoter and interacts with FliC and PilA, thus responding to influence swimming motility, twitching motility, and biofilm in *A. citrulli*. Therefore, *Ac*Δ*oxyR* displayed reduced virulence and ability to colonize melon seedlings by significantly affecting anti-oxidative stress as well as expression of flagellin and type IV pili-related gene (*fliC* and *pilA*). In the future, we will further investigate the global expression network of the *A. citrulli* OxyR.

## Data availability statement

The original contributions presented in the study are included in the article/Supplementary material; further inquiries can be directed to the corresponding authors.

## Author contributions

JW, JL, YZ, MS, and JF designed the experiments. JW, GY, YT, and BH performed the experiments and analyzed the data. JW and JL wrote the manuscript. YT and BH revised the manuscript and provided guidance for the experiments. All authors contributed to the article and approved the submitted version.

## Funding

This research was supported by National Key Research and Development Program (2021YFC2600602) and the Modern Agriculture Industrial Technology System Program of JiangSu, Grant/Award Number: JATS[2020]309.

## Conflict of interest

The authors declare that the research was conducted in the absence of any commercial or financial relationships that could be construed as a potential conflict of interest.

## Publisher’s note

All claims expressed in this article are solely those of the authors and do not necessarily represent those of their affiliated organizations, or those of the publisher, the editors and the reviewers. Any product that may be evaluated in this article, or claim that may be made by its manufacturer, is not guaranteed or endorsed by the publisher.

## Footnotes

^1^ http://www.softberry.com/berry.phtml?topic=bprom&group=programs&subgroup=gfindb
