# Supplementary material for: OxyR contributes to virulence of Acidovorax citrulli by regulating anti-oxidative stress and expression of flagellin FliC and type IV pili PilA
Source: Front Microbiol. 2022 Sep 20;13:977281. doi: 10.3389/fmicb.2022.977281 (PMC9530317; doi:10.3389/fmicb.2022.977281)
Supplement: Supplementary file 1 [file Table_1.docx]

**Table S1. Bacteria and plasmids used in this study.**

| **Bacteria strains** | **Relevant characteristics** | **Source** |
| --- | --- | --- |
| ***Acidovorax citrulli*** | | |
| xjl12 | Wild-type, Rif^R^ | This lab |
| *Ac*Δ*oxyR* | *oxyR* mutant strain, containing truncated *oxyR* gene and Km cassette,Rif^R^, Km^R^ | This study |
| *Ac*Δ*oxyR* (pBBR-OxyR) | *oxyR* complementation strain,containing pBBR-OxyR, Rif^R^, Km^R^,Gm^R^ | This study |
| *Ac*Δ*ahpC* | *ahpC* mutant strain, containing truncated *ahpC* gene and Km cassette,Rif^R^, Km^R^ | This study |
| *Ac*Δ*ahpC* (pBBR-AhpC) | *ahpC* complementation strain,containing pBBR-AhpC, Rif^R^, Km^R^,Gm^R^ | This study |
| *Ac*Δ*catB* | *catB* mutant strain, containing truncated *catB* gene and Km cassette,Rif^R^, Km^R^ | This study |
| *Ac*Δ*catB* (pBBR-CatB) | *catB* complementation strain,containing pBBR-CatB, Rif^R^, Km^R^,Gm^R^ | This study |
| WT(pBBR-CatB) | Wild-type xjl12 containing pBBR-CatB, Rif^R^ ,Gm^R^ | This study |
| *Ac*Δ*pilA* | *pilA* mutant strain, containing truncated *pilA* gene and Km cassette, Rif^R^, Km^R^ | This study |
| *Ac*Δ*pilA* (pBBR-PilA) | *pilA* complementation strain,containing pBBR-PilA, Rif^R^, Km^R^,Gm^R^ | This study |
| *Ac*Δ*fliC* | *fliC* mutant strain, containing truncated *fliC* gene and Km cassette, Rif^R^, Km^R^*fliC* | This study |
| *Ac*Δ*fliC* (pBBR-FliC) | *fliC* complementation strain,containing pBBR- FliC, Rif^R^, Km^R^,Gm^R^ | This study |
| WT(pBBR- *pilA*-Flag) | Wild-type xjl12 containing pBBR- *pilA*-Flag, Rif^R^, Gm^R^ | This study |
| *Ac*Δ*oxyR*(pBBR- *pilA*-Flag) | *Ac*Δ*oxyR* containing pBBR- *pilA*-Flag, Rif^R^, Km^R^,Gm^R^ | This study |
| WT(pBBR- *fliC*-Flag) | Wild-type xjl12 containing pBBR- *fliC*-Flag,Rif^R^, Gm^R^ | This study |
| *Ac*Δ*oxyR*(pBBR- *fliC*-Flag) | *Ac*Δ*oxyR* containing pBBR- *fliC*-Flag, Rif^R^, Km^R^,Gm^R^ | This study |
| ***Escherichia coli*** | | |
| DH5α | *Φ80 lacZΔM15,Δ(lacZYA-argF)U169.recA1, endA1.thi-1* | TaKaRa,  Dalian, China |
| S17-1(λpir) | *Λpir pro hsdR, recA* | Simon et al.  (1983) |
| XL1-Blue MRF´ Kan | D(mcrA)183, D(mcrCB-hsdSMR-mrr)173, endA1,supE44, thi-1, recA1 gyrA96,relA1, lac,  [F´proAB lacIqZDM15 Tn5 (KmR)] | (Wang et al., 2018) |
| **Plasmids** | | |
| pEX18GM | Suicide vector with a *sacB* gene, Gm^R^ | (Hoang et al.,1998) |
| pBBR1-MCS-5 | Broad host range vector, Gm^R^ | (Kovach et al.,1995) |
| pET30a | Containing kanamycin cassette, Km^R^ | This lab |
| pTRG | Plasmid used for protein expression in bacterial one-hybrid and bacterial two-hybrid assay, Tet^R^ | (Wang et al., 2018) |
| pBXcmT | Plasmid used for DNA cloning in bacterial one-hybrid assay, Chlo^R^ | (Wang et al., 2018) |
| pBT | Plasmid used for DNA cloning in bacterial two-hybrid assay, Chlo^R^ | (Han et al., 2020) |
| pMD19-T | ‘TA’ cloning vector | TaKaRa |
| pTRG-OxyR | pTRG with the coding region of OxyR, Tet^R^ | This study |
| pBXcmT-P_*fliC* | pBXcmT with the *fliC* promoter region, Chl^R^ | This study |
| pBXcmT-P_*pilA* | pBXcmT with the *pilA* promoter region, Chl^R^ | This study |
| pBT-FliC | pBT with the coding region of FliC, Chl^R^ | This study |
| pBT-PilA | pBT with the coding region of PilA, Chl^R^ | This study |

Rif^R^, Gm^R^, Km^R^, Tet^R^, Amp^R^ and Chl^R^ indicate resistance to Rifamycin, Gentamicin, Kanamycin, Tetracycline, Ampicilin and Chloromycetin, respectively.

**Table S2. Oligonucleotide primers used in this study.**

| Primer | Sequence(5’-3’) | Length of production |
| --- | --- | --- |
| **For mutant construction** | | |
| *oxyR*-F1 | GCTCTAGACGCCCATCGTCACCAAACTG | 864 |
| *oxyR*-R1 | CGGGATCCGTGCAACGGCGACGATGTAT |  |
| *oxyR*-F2 | CGGGATCCCACCCGCCTTTCCTGAGAAG | 720 |
| *oxyR*-R2 | GGGGTACCCATCGCATCGCAGGCAGTAC |  |
| *ahpC*-F1 | CCAAGCTTGCGATGCCTTCCTTGATGGT | 601 |
| *ahpC*-R1 | CGGGATCCGGCTGGACTTGGGTATTGAT |  |
| *ahpC*-F2 | GGGGTACCGCAAGATCTAATCGCCCGC | 361 |
| *ahpC*-R2 | CGGAATTCGTGAAGGACGGCTTGCGG |  |
| *catB*-F1 | GCTCTAGAGCATCATCAACATCGCCTCC | 648 |
| *catB*-R1 | GGGGTACCCGGTCACAAGTCTCTGGAAT |  |
| *catB*-F2 | GGGGTACCGCGCTGAAGGACTGATACCG | 603 |
| *catB*-R2 | CGGGATCCCCAGGTCCCGCAGAAGTT |  |
| *pilA*-F1 | CCAAGCTTCGTCGCTTCCATCACCAAG | 423 |
| *pilA*-R1 | GCTCTAGAGCCACCACGATCATCAGTTC |  |
| *pilA*-F2 | CGGAATTCGCGACACCATTCTCAAATTCC | 400 |
| *pilA*-R2 | GGGGTACCGCCATCGCCTAAGAATAGCA |  |
| *fliC*-F1 | CCAAGCTTATGGAGGGATTGTTGGGGAG | 339 |
| *fliC*-R1 | GCTCTAGAGAGGTGTTGAGCGAGGATTG |  |
| *fliC*-F2 | CGGGATCCGAAACCGCCAACCTGTCG | 325 |
| *fliC*-R2 | GGGGTACCAGCTCGCCATATACCACCTC |  |
| Km-F | GAAGCTCCCTCGTGC | 1514 |
| Km-R | CAGGTGGCACTTTTCG |  |
| **For complementary strains construction** | | |
| *oxyR-*comp-F | GGGGTACCGTGATCGAGGTGGGCGTG | 1762 |
| *oxyR-*comp-R | GCTCTAGAGTAGAGCGTGTAGGTGTCGG |  |
| *oxyR-*flag-F | GGGGTACCCGTGCTGCTCTATTCGACG | 1409 |
| *oxyR-*flag-R | GCTCTAGATCACTTATCGTCGTCATCCTTGTAATCGGAAAGGCGGGTGACGC |  |
| *ahpC-*comp-F | GGGGTACCTAATGGAATGGTGCGGTGTC | 1005 |
| *ahpC-*comp-R | GCTCTAGAGGCGGAAAGTTGGGATTTGA |  |
| *ahpC-*flag-F | CCAAGCTTTAATGGAATGGTGCGGTGTC | 840 |
| *ahpC-*flag-R | GCTCTAGATTACTTATCGTCGTCATCCTTGTAATCGATCTTGCCGACCAGGT |  |
| *catB-*comp-F | GGGGTACCTACGGAGATCAACCACCACC | 2276 |
| *catB-*comp-R | GCTCTAGAAACTCGCCCACCATGTCC |  |
| *catB-*flag-F | CCAAGCTTTACGGAGATCAACCACCACC | 2011 |
| *catB-*flag-R | GCTCTAGATCACTTATCGTCGTCATCCTTGTAATCGTCCTTCAGCGCGGCGG |  |
| *pilA-*comp-F | CCAAGCTTACGCGGTGGAGATCGAAG | 1238 |
| *pilA-*comp-R（flag） | GCTCTAGATTACTTATCGTCGTCATCCTTGTAATCAGGAGCAAA |  |
| *fliC-*comp-F | GGGGTACCGCTGATGTTGGTGTTGATGG | 1867 |
| *fliC-*comp-R(flag) | GCTCTAGATCACTTATCGTCGTCATCCTTGTAATCACGCAGCAGGGACAGCACGCCCT |  |
| **For qRT-PCR** | | |
| *16s*-F | CCTACGGGAGGCAGCAG | 177 |
| *16s*-R | ATTACCGCGGCTGCTGG |  |
| *oxyR*-F | GGCTCGTCGCTCGAAACCAT | 139 |
| *oxyR*-R | GGTAGCGGATGTGGGTGTCG |  |
| *ahpC*-F | GAAGACGCCGCCGACAACTA | 171 |
| *ahpC*-R | GAAGGCGTTGGTCAGCTGGT |  |
| *catB*-F | ACGACCTGGTGAACGCCATC | 157 |
| *catB* -R | ACCATCTGGCCGATCTTGCG |  |
| *pilA*-F | GAACTGATGATCGTGGTGGC | 168 |
| *pilA*-R | CGGAGGACTCGAAGCAGTAG |  |
| *fliC*-F | CCGCAACGTCTCGGAACTGA | 163 |
| *fliC*-R | CTGCGAGGACACGTCGTTGA |  |
| *fliS*-F | GTTGAGCACGGCCCTCGAT | 122 |
| *fliS*-R | CCAGCATTGCGTCGTCGTTC |  |
| *flgM*-F | CCGAAGAGGCCACCAAGACC | 117 |
| *flgM*-R | CATCGAAATCCGCGCTGCTG |  |
| *hrcC*-F | CCCGGTGGATGGCAAGAACA | 171 |
| *hrcC*-R | TCCGATTCGACGGTGATGCC |  |
| *hrcN*-F | CGTGGGCAAGAGTACGCTGA | 162 |
| *hrcN*-R | GAGGTGGCGCAGACCACTAC |  |
| *hrcQ*-F | CGAAGTGGCGGAAACCCTGA | 125 |
| *hrcQ*-R | CGCCCTGGGTCCATTCCAG |  |
| *hrpG*-F | CTGGGCCTGATCCGAAGCTG | 189 |
| *hrpG*-R | CGCGGTTTCCAGCGCATAG |  |
| **For bacterial one-hybrid and two-hybrid strains construction** | | |
| PTOxyR-F | AAGAATGCGGCCGCATGACCCTCACAGAACTCAA | 966 |
| PTOxyR-R | GGACTAGTTCAGGAAAGGCGGGTGACGC |  |
| P_*fliC*-F | CGGAATTCGCTGATGTTGGTGTTGATGG | 344 |
| P_*fliC*-R | GCTCTAGACTGACTGGATATGGCGTTGC |  |
| P_*pilA*-F | CGGAATTCAGCACCAACGGCACCTAT | 333 |
| P_*pilA*-R | GCTCTAGA CTTGGTGATGGAAGCGACG |  |
| pBTFliC-F | CGGGATCCATGGCTTCCACCATCAACAC | 1479 |
| pBTFliC-R | CCGCTCGAGTCAACGCAGCAGGGACAGCA |  |
| pBTPilA-F | CGGGATCCATGAAGCGTACTGTTCAGCA | 507 |
| pBTPilA-R | CCGCTCGAGTTAAGGAGCAAACGTGCCAG |  |

^a^ Base sequences underlined indicated the restriction enzyme sites.


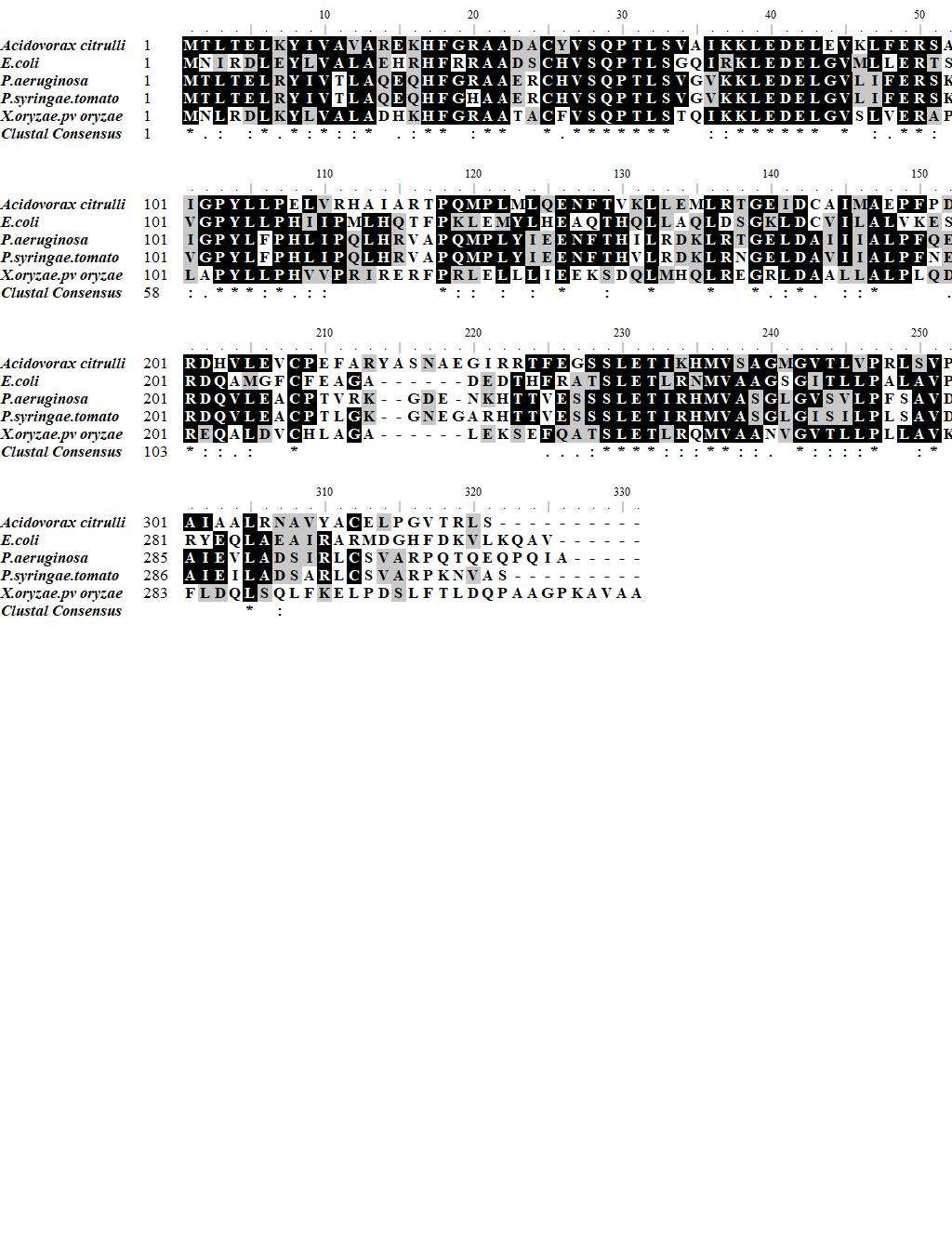


**Figure S1.** Multiple alignment of OxyR amino acid sequences in Acidovorax citrulli and other important bacteria including *E. coli*, *Pseudomonas aeruginosa*, *P. syringae* pv. *tomato* and *Xanthomonas* *oryzae* pv. *oryzae*. Conserved amino acids are shaded in black and homologous residues are shaded in gray.


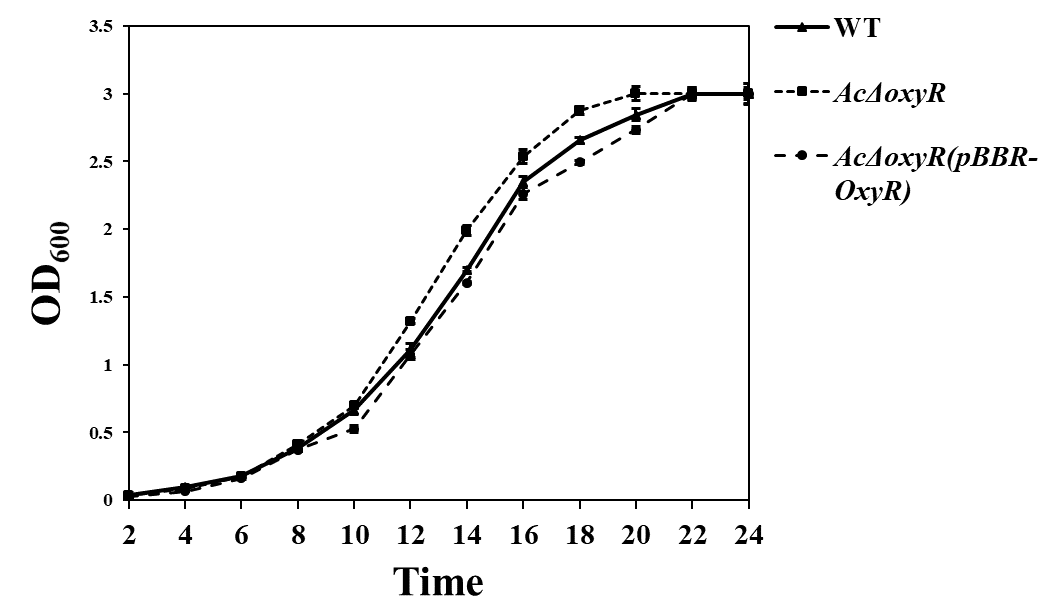


**Figure S2.** Population dynamics of *Acidovorax citrulli* wild-type (WT), *Ac*Δ*oxyR*, and *Ac*Δ*oxyR* (pBBR-OxyR) strains in Luria-Bertani (LB) broth at 28℃. The experiments were performed in triplicate and repeated three times.


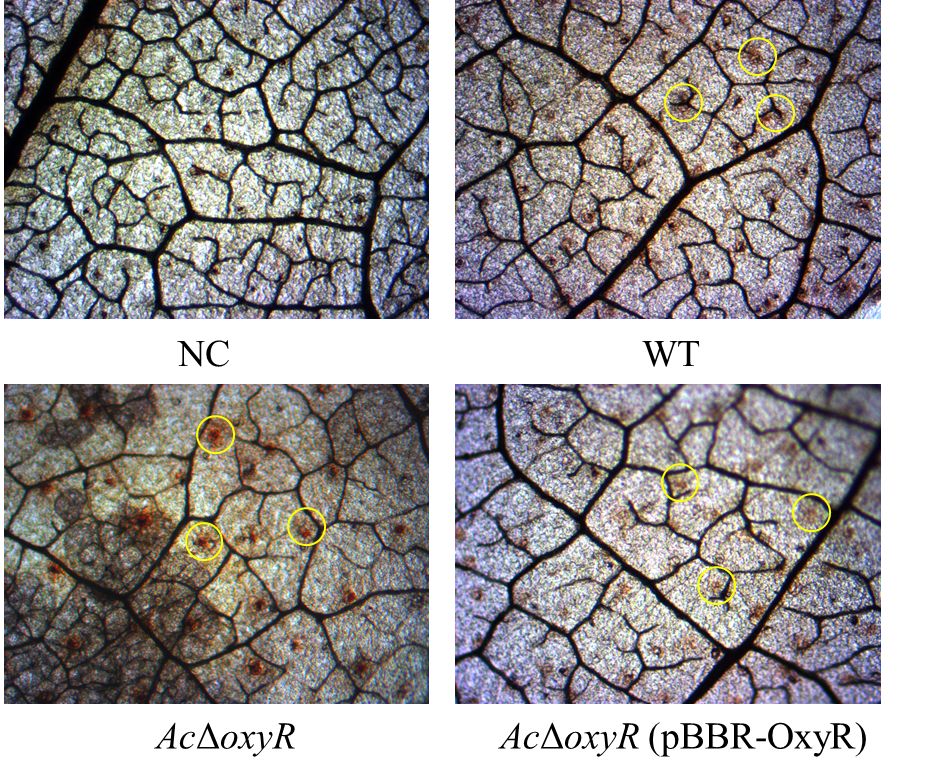


**Fig S3.** Histochemical detection of H_2_O_2_ by DAB staining in melon leaves. The melon plants were grown for one week and inoculated with ddH_2_O control (NC), *Acidovorax citrulli* wild-type (WT), *Ac*Δ*oxyR*, and *Ac*Δ*oxyR* (pBBR-OxyR). The red spots in the yellow circle indicate the presence of H_2_O_2_. The experiment was repeated three times.


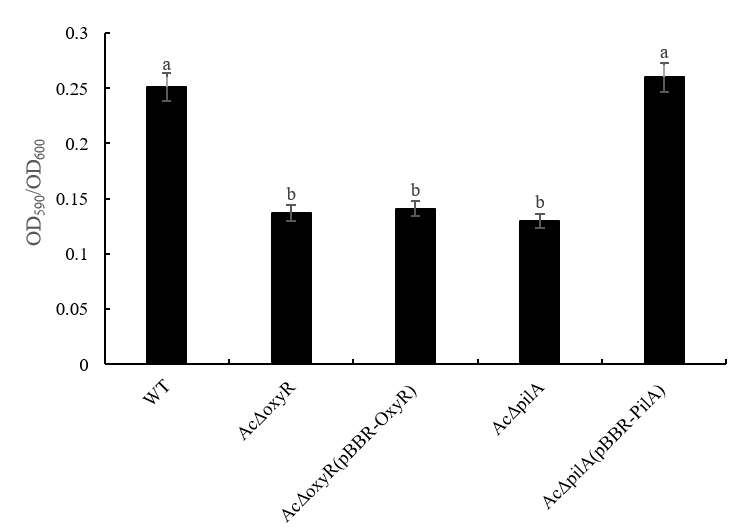


**Fig S4.** Quantitative analysis of biofilm production by WT, *Ac*Δ*oxyR*, *Ac*Δ*oxyR* (pBBR-OxyR), *Ac*Δ*pilA*, and *Ac*Δ*pilA* (pBBR-PilA) strains of *A. citrulli*. Optical density at 600 nm (OD_600_) was measured for after 48-h liquid cultures of each strain in 12-plate wells and biofilm production were measured after staining with crystal violet using a spectrophotometer at OD_590_/OD_600_. Different lowercase letters indicate a significant difference between treatments. Statistically significant differences were determined by the one-way ANOVA of variance and *p* < 0.05.


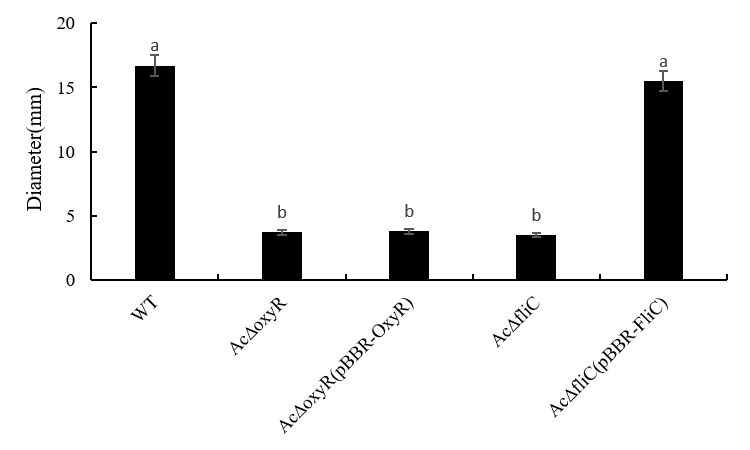


**Fig S5.** Quantitative analysis of swimming motility by WT, *Ac*Δ*oxyR*, *Ac*Δ*oxyR* (pBBR-OxyR), *Ac*Δ*fliC*, and *Ac*Δ*fliC* (pBBR-FliC) strains of *A. citrulli*. The diameters of swimming halos were measured after 3 days of incubation. Different lowercase letters indicate a significant difference between treatments. Statistically significant differences were determined by the one-way ANOVA of variance and *p* < 0.05.
